# Effect of survey instrument on participation in a follow-up study: a randomization study of a mailed questionnaire versus a computer-assisted telephone interview

**DOI:** 10.1186/1471-2458-12-579

**Published:** 2012-07-31

**Authors:** Carissa M Rocheleau, Paul A Romitti, Stacey Hockett Sherlock, Wayne T Sanderson, Erin M Bell, Charlotte Druschel

**Affiliations:** 1 Department of Epidemiology, College of Public Health, The University of Iowa, Iowa City, IA, 52242, USA; 2 National Institute for Occupational Safety and Health, Cincinnati, OH, 45226, USA; 3 Department of Occupational and Environmental Health, College of Public Health, The University of Iowa, Iowa City, IA, 52242, USA; 4 Department of Epidemiology and Biostatistics, School of Public Health, State University of New York at Albany, Rensselaer, NY, 12144, USA; 5 New York State Department of Health, Troy, NY, 12180, USA

## Abstract

**Background:**

Many epidemiological and public health surveys report increasing difficulty obtaining high participation rates. We conducted a pilot follow-up study to determine whether a mailed or telephone survey would better facilitate data collection in a subset of respondents to an earlier telephone survey conducted as part of the National Birth Defects Prevention Study.

**Methods:**

We randomly assigned 392 eligible mothers to receive a self-administered, mailed questionnaire (MQ) or a computer-assisted telephone interview (CATI) using similar recruitment protocols. If mothers gave permission to contact the fathers, fathers were recruited to complete the same instrument (MQ or CATI) as mothers.

**Results:**

Mothers contacted for the MQ, within all demographic strata examined, were more likely to participate than those contacted for the CATI (86.6% vs. 70.6%). The median response time for mothers completing the MQ was 17 days, compared to 29 days for mothers completing the CATI. Mothers completing the MQ also required fewer reminder calls or letters to finish participation versus those assigned to the CATI (median 3 versus 6), though they were less likely to give permission to contact the father (75.0% vs. 85.8%). Fathers contacted for the MQ, however, had higher participation compared to fathers contacted for the CATI (85.2% vs. 54.5%). Fathers recruited to the MQ also had a shorter response time (median 17 days) and required fewer reminder calls and letters (median 3 reminders) than those completing the CATI (medians 28 days and 6 reminders).

**Conclusions:**

We concluded that offering a MQ substantially improved participation rates and reduced recruitment effort compared to a CATI in this study. While a CATI has the advantage of being able to clarify answers to complex questions or eligibility requirements, our experience suggests that a MQ might be a good survey option for some studies.

## Background

Survey research faces two primary challenges: reaching potential subjects, and having potential subjects agree to participate. In the face of rapid societal change, survey methods must constantly be reexamined: new technology, security fears, and laws regarding communications technology may drastically change survey research [1-5]. Survey researchers must therefore adapt their techniques to changing circumstances in order to collect valid and reliable information about a representative sample of individuals.

Though once considered a major breakthrough in survey research, the utility of computer-assisted telephone interviews (CATIs) are challenged by new telephone technology and increasing use of wireless (cellular) telephones instead of (or in addition to) landlines. Caller ID and answering machines/voicemail have made it easier for people to screen their calls, making it more difficult for study staff to reach potential participants by telephone [4]. While methods for telephone sampling of landlines are well-established [6,7], sampling wireless numbers faces numerous challenges: numbers are portable, and may not be representative of a geographic region; there are no comprehensive public lists of wireless telephone numbers; federal laws prohibit use of automated dialers when calling wireless telephones; households with wireless telephones and landlines overlap; subjects generally pay for wireless minutes used; and subjects may be in public places when researchers call them, raising privacy considerations [2,4,5].

As wireless telephone usage increases, challenges to telephone-based surveys become more pronounced. Between 2003 and 2009, the number of U.S. households with wireless-only telephones increased from 6.7% to 24.5% [8,9]. Almost half of young adults, aged 25-29, live in wireless-only households [9]. Urban areas and households living in poverty also have a higher proportion of wireless-only households [8,9]. Demographic differences between people living in wireless-only versus landline households could cause bias in telephone surveys. Target populations with a low prevalence of landlines (e.g. young, urban adults) may also be hard for researchers to access in telephone surveys [2,10].

Given these increasing challenges to telephone surveys, some researchers are reevaluating the utility of mailed questionnaires (MQs). Addresses are often easier to obtain than telephone numbers and are geographically defined. MQs can be answered at a participant’s convenience. Several studies conducted from the 1970s through 1990s found that response rates for mailed questionnaires were lower than for telephone surveys [11-13]. Due to changing trends in telephone usage and declining response rates [4,14], these studies may be less relevant to current survey research. A more recent randomized study of survey instruments showed improved participation rates for those who received a MQ versus a CATI [15]. In that study, the difference was most pronounced for women and younger adults, with women in their 30s being 19% more likely to complete a MQ than a CATI [15].

In comparing instruments, however, it is important to separate the effects of the survey mode from the effects of the recruitment methods. To determine the best survey mode for participation in a follow-up study to a large, multisite population-based study, we conducted a pilot study in which families were randomized to be recruited to complete either a mailed questionnaire (MQ) or CATI using similar recruitment protocols. The purpose of this pilot was to determine whether survey instrument (MQ or CATI) impacted participation rates in a subset of respondents to a previous telephone survey. The pilot also aimed to compare the response rates of mothers and fathers using both survey instruments, and to investigate the representativeness of participants for each instrument.

## Methods

### National birth defects prevention study

Mothers of case or control infants who had completed participation in the National Birth Defects Prevention Study (NBDPS) were recruited. The NBDPS is an ongoing population-based case-control study of birth defects conducted in 10 states in the US (Arkansas, California, Iowa, Georgia, Massachusetts, New Jersey, New York, North Carolina, Texas, and Utah); details have been described elsewhere [16]. The NBDPS protocols are approved by the Human Subjects Review Board of the Centers for Disease Control and Prevention and each of the state-based centers. Mothers of cases and non-malformed controls completed telephone interviews of their medical and reproductive histories, lifestyle factors, and pregnancy characteristics. At the time of the NBDPS interview, contact information (both address and telephone number) was updated for all participants.

### Home and occupational exposure to pesticides study

As a follow-up study to the NBDPS, a sample of participants was contacted and asked to participate in the Home and Occupational Exposure to Pesticides Study (HOEPS). Eligible mothers were those from the Iowa and New York NBDPS centers who had pregnancies ending between January 1, 2004 and December 31, 2005 and who had already completed participation in the NBDPS. All mothers of infants diagnosed with either hypospadias or a heart defect were recruited, as previous studies have suggested an association between pesticides and these defects [17,18], and mothers of these cases were not enrolled in other follow-up studies. We attempted to recruit one control per case. The HOEPS pilot protocol was approved by the Institutional Review Boards at the University of Iowa and the New York State Department of Health.

Recruitment for the HOEPS occurred from June 2007 to June 2008. Because only mothers participate in the NBDPS interview, recruitment for the HOEPS was initiated with mothers; biological fathers were not contacted for the HOEPS unless permission was given by mothers. Eligible case and control mothers were randomly assigned to be recruited with either a MQ or CATI: each mother was assigned a random number using the RAND function in Microsoft® Excel 2003 (Microsoft Corporation, USA), records were ordered by the assigned random number, and the lower and upper halves were assigned to the MQ and CATI instrument arms, respectively.

Recruitment followed detailed protocols. In order to isolate the effect of survey instrument from the effects of recruitment methods, the recruitment materials and protocols were designed to provide as comparable an experience as possible to those recruited to the MQ and CATI while using mixed-mode recruitment materials (both telephone and mailed). The only differences in the content of the recruitment materials were instructions/materials that were specific to the survey mode. All mailings were sent via the US Postal Service with address service requested. Where the recorded address or telephone number was no longer accurate, we attempted tracing using a combination of online and/or public data (telephone/address directories, driver license registration, and voter registration), patient contact records, and commercial tracing services. If current contact information could not be identified for a mother, the family was excluded.

Each mother whose contact information could be obtained was initially sent a letter notifying her that we were conducting a follow-up study and would soon be sending out a packet of information about the study. Mothers were given an “address and telephone update sheet” and a pre-addressed, stamped return envelope. Regardless of whether the address update was received, an introductory packet was sent 2 weeks later that included financial compensation ($20 check). For those randomized to the MQ, a questionnaire and pre-addressed stamped envelope were included in the introductory packet. For those randomized to the CATI, a form indicating preferred times and days to call was enclosed as well as a pre-addressed stamped return envelope; mothers were also given a call-in telephone number if they wished to schedule the interview.

Reminders began after 1 week for those randomized to the CATI and after 2 weeks for those randomized to the MQ (to allow adequate time to review, complete, and return the questionnaire). We attempted up to 3 reminder cycles, two weeks apart, consisting of a telephone reminder followed by a mailed reminder. Because we hoped to deliver a telephone reminder personally, we attempted calling up to 4 times (morning, afternoon, evening, and weekend) for each telephone reminder; we left a message only once (typically on the last call attempt). If we reached the potential participant during any call attempt, the telephone reminder was considered complete. If we did not hear back from potential participants (after 3 days for those assigned to the CATI or 7 days for those assigned to the MQ, to allow for mailing time), we sent a reminder letter. If no response was received after two weeks, the next reminder cycle began.

**Figure 1:**
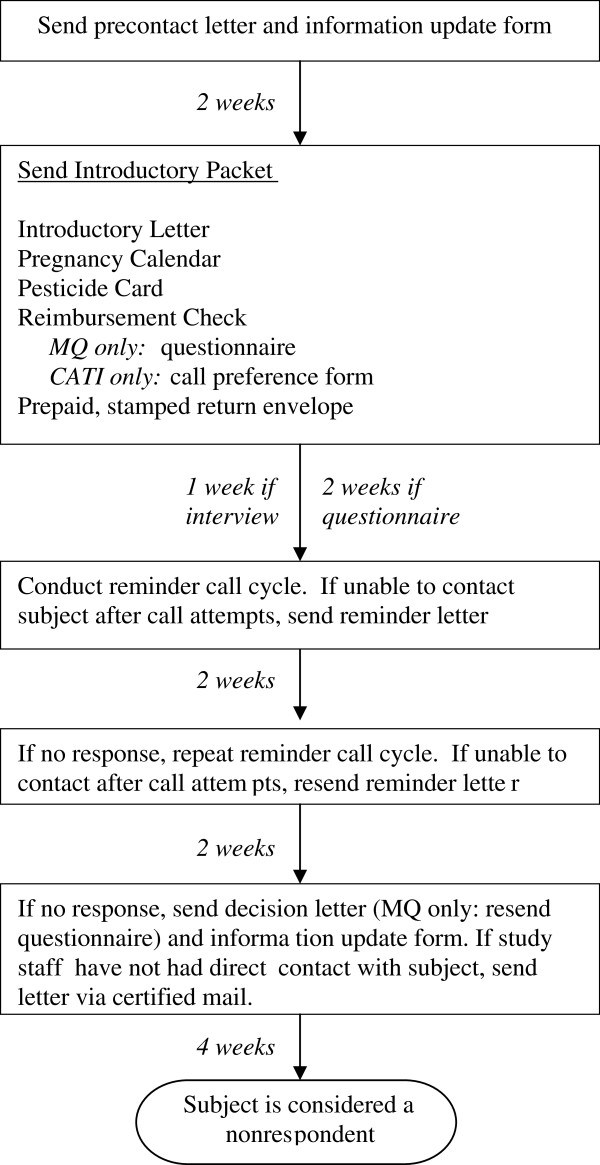
General recruitment protocol for the HOEPS pilot.

During the third (final) cycle, the reminder letter informed potential participants that this would be their final contact. For those assigned to the MQ, a copy of the questionnaire and pre-addressed stamped return envelope were enclosed. Those assigned to the CATI were given a form (indicating preferred times and days to be called) and pre-addressed stamped envelope to return if they still wished to participate; they were also provided with a call-in number. If study staff never had direct contact with a potential participant, the decision letter was sent via certified mail. Those who did not respond to the final decision letter were considered non-respondents (Figure 1). For parents who indicated their intention to participate (by scheduling a telephone interview, returning the CATI decision form, or requesting that a new questionnaire be mailed), we made additional attempts at contact.

The survey instrument covered the six months prior to conception through the end of pregnancy (B6-T3). The maternal and paternal surveys contained a module on occupational pesticide exposure; the maternal survey also included modules to record residential pesticide treatments, environmental pesticide exposure, and when/if the father resided in the same residence during the survey period. Both the MQ and CATI asked the same questions; however some changes in formatting (such as arranging questions into a table format on the MQ) were made when appropriate for the survey mode. On the last page of the MQ or at the completion of the CATI, mothers were asked for permission to contact the biological father of the index child. If permission was given, contact information for the father was requested (if known). Fathers were assigned to the same instrument (MQ or CATI) as mothers, and were recruited with the same protocol used for mothers.

### Statistical analysis

All analyses were conducted with SAS 9.1.3 (SAS Institute Inc, Cary, NC, USA). Descriptive analyses examined participation rates by case or control status and type of instrument (MQ or CATI); statistical significance (p < 0.05) of the difference in participation rates was calculated using Fisher’s Exact test. Bivariable analyses were used to determine demographic characteristics of families participating in the HOEPS, as well as potentially eligible families from the NBDPS, stratified by instrument. For both mothers and fathers, we calculated the median number of days between mailing of the introductory packet and return of a completed questionnaire /completion of an interview and median number of reminders (both telephone and mailed) after mailing the introductory packet, stratified by instrument. The Wilcoxon rank-sum test was used to determine whether the difference in median number of days or number of reminders was statistically significant between instruments (p < 0.05).

## Results

A total of 392 women were eligible for the HOEPS; a current address or telephone number was not identified for 51 (13%, Figure 1). Contact information could not be identified for more mothers of controls compared to cases (16.3% vs. 9.5%). In total, 341 women were invited to participate in the HOEPS and 268 (68.4%) completed participation. Among these women, 214 (79.9%) gave permission to contact the index child’s biological father. Valid contact information could not be identified for 5 of these fathers, resulting in 209 fathers who were invited to participate in the HOEPS. Of these, 147 (54.9% of all fathers) completed participation (Table 1).

**Table 1:** Recruitment status of families eligible for the HOEPS pilot

	** *N (%)* **	** *N (%)* **	** *N (%)* **
	**Controls**	**Cases**^a^	**Total**
**Final Status of Eligible Mothers**	**(n = 203)**	**(n = 189)**	**(n = 392)**
Excluded: no valid contact information	33 (16.3)	18 (9.5)	51 (13.0)
Invited to participate in HOEPS	170 (83.7)	171 (90.5)	341 (87.0)
Refused participation	18 (8.9)	15 (7.9)	33 (8.4)
Non-response with contact ^b^	20 (9.9)	17 (9.0)	37 (9.4)
Non-response without contact^b^	2 (1.0)	1 (0.5)	3 (0.8)
Completed participation	130 (64.0)	138 (73.0)	268 (68.4)
	**Controls**	**Cases**^c^	**Total**
**Final Status of Eligible Fathers**	**(n = 130)**	**(n = 138)**	**(n = 268)**
Mother did not give permission to contact	37 (28.5)	17 (12.3)	214 (79.9)
Excluded: no valid contact information	3 (2.3)	2 (1.4)	5 (1.9)
Invited to participate in HOEPS	90 (69.2)	119 (86.2)	209 (78.0)
Refused participation	8 (6.2)	4 (2.9)	12 (4.5)
Non-response with contact ^b^	16 (12.3)	34 (24.6)	50 (18.7)
Non-response without contact ^b^	0 (0)	0 (0)	0 (0)
Completed participation	66 (50.8)	81 (58.7)	147 (54.9)

^a^ Cases include 164 mothers of infants with congenital heart defects and 25 mothers of infants with hypospadias.

^b^ Contact is defined as speaking with study staff over the telephone, returning a completed call preference form or information update form, or personally signing for a decision letter sent via certified mail. Contact confirms that the eligible individual received recruitment materials.

^c^ Cases include 109 eligible fathers of infants with congenital heart defects and 12 eligible fathers of infants with hypospadias.

Participation rates by instrument and case/control status (Table 2) were calculated only among those who were sent an invitation to participate in HOEPS (i.e., excluding those whose contact information we could not obtain, and fathers we were not given permission to contact). Among the 341 mothers invited to participate in the HOEPS, mothers of case and control infants had similar rates of participation (80.7% vs. 76.5%; p = 0.36; data not shown).Fathers of the 209 case and control infants who were invited to participate in the HOEPS also had similar participation rates (68.1% vs. 73.3%, p = 0.45; data not shown). Mothers recruited to the MQ, however, were more likely to participate than those recruited to the CATI (86.6% vs. 70.6% respectively; p < 0.001) (Table 2). Among case mothers, participation rates were higher for those assigned to the MQ (86.9%) versus those assigned to the CATI (74.7%); there was an even larger difference in participation rates between control mothers recruited to the MQ (86.2%) versus the CATI (66.3%) (Table 2).

**Table 2:** Participation of parents invited to participate in the HOEPS pilot, by survey instrument and case or control status

	**MQ**			**CATI**			**TOTAL**
	**Cases**	**Controls**	**Total**	**Cases**	**Controls**	**Total**	**Eligible**
	**(n = 84)**	**(n = 87)**	**(n =171)**	**(n = 87)**	**(n = 83)**	**(n =170)**	**(n =341)**
	** *N (%)* **	** *N (%)* **	** *N (%)* **	** *N (%)* **	** *N (%)* **	** *N (%)* **	** *N (%)* **
Mother participated^a^	73 (86.9)	75 (86.2)	148 (86.6)	65 (74.7)	55(66.3)	120 (70.6)	268 (78.6)
Permission to contact father^a,b^	60 (82.2)	51 (68.0)	111 (75.0)	61 (93.9)	42(76.4)	103 (85.8)	214 (79.9)
Father participated^a,c^	49 (83.1)	43 (87.8)	92 (85.2)	32 (53.3)	23(56.1)	55 (54.5)	147 (70.3)
Overall father participation^a,d^	49 (58.3)	43 (49.4)	92 (53.8)	32 (36.8)	23(27.7)	55 (32.4)	147 (43.1)

^a^ difference between CATI and MQ was significant (p-value <0.05).

^b^ the denominator for permission rate is the number of mothers who participated.

^c^ among fathers that project staff were given permission to contact.

^d^ among all fathers potentially eligible for participation at the beginning of HOEPS. Fathers were lost to participation when the mother did not participate, when the mother did not give permission to contact the father, when contact information could not be found for the father, and when the father chose not to participate.

**Figure 2:**
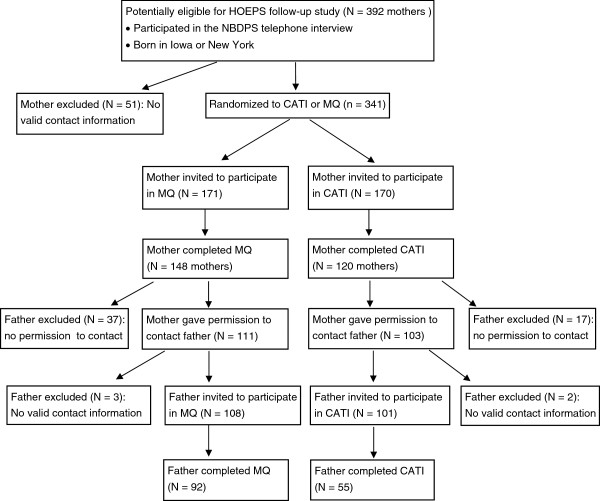
Participation and recruitment of mothers and fathers for the HOEPS pilot.

Cohabitating with the biological father during the six months prior to conception through the end of pregnancy was reported by 86.2% of participating mothers. Mothers who reported living with the child’s biological father during this time period were more likely to give permission to contact the father, compared to mothers who did not live with the father for all of that time period (86.2% vs. 48.65%, p < 0.001; data not shown). Mothers who completed the MQ were less likely to give permission to contact the father compared to mothers who completed the CATI (75.0% vs. 85.8% respectively; p = 0.03; Table 2). Fathers invited to complete the MQ, however, had higher participation rates than those invited to complete the CATI (85.2% vs. 54.5%, Table 2); consequently more fathers participated with the MQ overall (n = 92) compared to the CATI (n = 55, Figure 2).

The distribution of demographic and pregnancy characteristics (maternal age, race, education, smoking status, parity, family income, and infant birthweight) was similar between mothers who participated in HOEPS and eligible mothers from the NBDPS (Table 3). Within every demographic strata we examined, participation rates were higher for mothers assigned to the MQ versus mothers assigned to the CATI (Table 3).

**Table 3:** Participation of parents invited to participate in the HOEPS pilot, by survey instrument and case or control status

	**Distribution of demographic characteristics**				**Participation rate**	
	**Eligible**		**Participated**			
	**MQ**	**CATI**	**MQ**	**CATI**	**MQ**	**CATI**
	**(n = 195)**	**(n = 197)**	**(n = 148)**	**(n = 120)**	**(n = 148)**	**(n = 120)**
	** *N (%)* **	** *N (%)* **	** *N (%)* **	** *N (%)* **	** *%* **	** *%* **
Case status						
Cases	94 (48.2)	95 (48.2)	73 (49.3)	65 (54.2)	77.7	68.4
Controls	101 (51.8)	102 (51.8)	75 (50.7)	55 (45.8)	74.3	53.9
Center						
Iowa	123 (63.1)	123 (62.4)	92 (62.2)	78 (65.0)	74.8	63.4
New York	72 (36.9)	74 (37.6)	56 (37.8)	42 (35.0)	77.8	56.8
Mother’s age at conception						
<20 years	17 (8.7)	15 (7.6)	10 (6.8)	7 (5.8)	58.8	46.7
20-24 years	49 (25.1)	45 (22.8)	36 (24.3)	24 (20.0)	73.5	53.3
25-29 years	56 (28.7)	60 (30.5)	42 (28.4)	40 (33.3)	75.0	66.7
30-34 years	49 (25.1)	57 (28.9)	39 (26.4)	34 (28.3)	79.6	59.6
≥35 years	24 (12.3)	20 (10.2)	21 (14.2)	15 (12.5)	87.5	75.0
Mother’s race/ethnicity						
White, non-Hispanic	171 (87.7)	176 (89.3)	134 (90.5)	108 (90.0)	78.4	61.4
Other race	21 (10.8)	21 (10.7)	13 (8.8)	12 (10.0)	61.9	57.1
Missing/Unknown	3 (1.5)	0 (0.0)	1 (0.7)	0 (0.0)	NC	NC
Mother’s education						
<12 years, or did not complete high school (HS)	12 (6.2)	11 (5.6)	6 (4.1)	5 (4.2)	50.0	45.5
12 years, completed HS, or equivalent	38 (19.5)	43 (21.8)	27 (18.2)	25 (20.8)	71.1	58.1
1-3 years college, or completed technical college	70 (35.9)	68 (34.5)	53 (35.8)	37 (30.8)	75.7	54.4
≥4 years of college; bachelor's degree or higher	75 (38.5)	75 (38.1)	62 (41.9)	53 (44.2)	82.7	70.7
Family’s annual income						
<$20,000	36 (18.5)	42 (21.3)	24 (16.2)	22 (18.3)	66.7	52.4
$20,000 - $39,999	53 (27.2)	52 (26.4)	39 (26.4)	32 (26.7)	73.6	61.5
≥$40,000	100 (51.3)	98 (49.7)	80 (54.1)	63 (52.5)	80.0	64.3
Missing	6 (3.1)	5 (2.5)	5 (3.4)	3 (2.5)	83.3	60.0
Mother smoked						
Yes	56 (28.7)	49 (24.9)	37 (25.0)	21 (17.5)	66.1	42.9
No	139 (71.3)	147 (74.6)	111 (75.0)	98 (81.7)	79.9	66.7
Missing	0 (0.0)	1 (0.5)	0 (0.0)	1 (0.8)	NC	NC
Parity						
Primiparous	87 (44.6)	69 (35.0)	67 (45.3)	41 (34.2)	77.0	59.4
Multiparous	108 (55.4)	127 (64.5)	81 (54.7)	78 (65.0)	75.0	61.4
Missing	0 (0.0)	1 (0.5)	0 (0.0)	1 (0.8)	NC	NC
Infant birthweight						
<2500 g	21 (10.8)	24 (12.2)	15 (10.1)	9 (7.5)	71.4	37.5
≥2500 g	172 (88.2)	170 (86.3)	132 (89.2)	109 (90.8)	76.7	64.1
Missing	2 (1.0)	3 (1.5)	1 (0.7)	2 (1.7)	NC	NC

*NC* not calculated.

After receiving their introductory packets, the median time for mothers completing and returning the mailed questionnaire was 17 days; this was significantly (p < 0.05) shorter than the median time for completing the CATI (29 days, Table 4). Mothers assigned to the MQ also required significantly fewer reminders (median of 3 reminders versus 6, p < 0.05). Fathers participating in the MQ also completed their surveys in less time compared to fathers participating in the CATI (median 28 days vs. 17 days; p = 0.09), and required fewer reminders (median 3 vs. 6; p < 0.001) (Table 4). There was no significant difference between survey modes in time between birth of the index child and completion of the HOEPS (Table 3).

**Table 4:** Response characteristics of participants in HOEPS pilot, by survey instrument and case or control status

	**Cases Median (IR)**	**Controls Median (IR)**	**Total Median (IR)**
Mothers	N = 138	N = 130	N = 268
Time to completion (days) ^a^			
CATI	29.0 (15.0 – 56.0)	29.0 (13.0 – 43.0)	29 (14.0 - 51.5)
MQ	20.0 (13.0 – 35.0)	17.0 (9.0 – 39.0)	17 (10.0 - 35.0)
Number of reminders ^a,b^			
CATI	6.0 (4.0 – 9.0)	6.0 (4.0 – 8.0)	6.0 (4.0 - 8.0)
MQ	3.0 (2.0 – 5.0)	3.0 (2.0 – 4.0)	3.0 (2.0 - 4.0)
Time elapsed from child’s birth to completion of HOEPS (years)			
CATI	2.9 (2.2 - 3.2)	2.6 (2.1 - 3.2)	2.8 (2.2 - 3.2)
MQ	2.7 (2.3 - 3.3)	2.6 (2.1 - 3.2)	2.6 (2.2 - 3.3)
Fathers	N = 81	N = 66	N = 147
Time to completion (days)			
CATI	28.0 (14.0- 53.5)	21.0 (16.0 – 42.0)	28.0 (14.0 - 49.0)
MQ	20.0 (13.0- 41.0)	16.0 (10.0 – 41.0)	17.0 (10.0 - 41.0)
Number of reminders ^a,b^			
CATI	6.0 (4.0 - 6.5)	5.0 (4.0 – 7.0)	6.0 (4.0 - 7.0)
MQ	3.0 (1.0 - 5.0)	2.0 (1.0 – 5.0)	3.0 (1.0 - 5.0)
Time elapsed from child’s birth to completion of HOEPS (years)			
CATI	3.1 (2.6 - 3.4)	2.6 (2.3 – 3.5)	3.0 (2.4- 3.5)
MQ	2.9 (2.4 – 3.4)	2.7 (2.3 – 3.2)	2.8 (2.3- 3.3)

*IR* interquartile range.

^a^ difference between CATI and MQ was statistically significant (p < 0.05).

^b^ This includes reminder letters and calls; a reminder call consists of up to 4 call attempts. Participants who returned the MQ without a reminder, or who called in to schedule/complete an interview after receiving the introductory packet, had 0 reminders.

## Discussion

Overall, participation among recruited mothers was good in the HOEPS. Fathers invited to participate in the HOEPS also had high participation rates, unlike a previous study that found women were 10% more likely to complete an initial survey than men [19]. Fathers were lost to participation, however, when the mother could not be located, refused participation in HOEPS, or did not give permission to contact the father. Information provided from fathers directly may be more accurate than those reported by proxy, however [20-24]. Our results show that obtaining information from fathers, in addition to mothers, is feasible though participation rates may be lower when mothers are ‘gatekeepers’ to study participation. In studies of reproductive outcomes, mothers are often contacted first: mothers are the sources of information (or permission to medical records) about pregnancy and delivery characteristics, fathers may not be specified on birth records, and biological fathers may not be aware of the birth of the child. Since mothers who resided with the index child’s biological father six months before and during the index pregnancy were more likely to give permission for researchers to contact the father, this could be an important source of selection bias in evaluating paternal exposures in studies of birth outcomes where mothers act as gatekeepers.

Fewer mothers completing the MQ gave permission to contact fathers compared to mothers completing the CATI; this was not explained by underlying differences in the percentages of mothers who reported living with their child’s biological father during the entirety of B6-T3. Mothers who received the MQ had the opportunity to ask fathers if they wanted to be contacted before giving us permission; this may explain why mothers assigned to the MQ tended to give permission less often to contact the father, but the fathers were more likely to participate when contacted. During the CATI, mothers also had an opportunity to clarify what was expected of the father (including what information would be solicited and how long the interview would take). Sending both the maternal and paternal MQs to the mother at the same time might increase paternal participation; mothers could then choose whether or not to pass the paternal MQ to the father immediately if they share a residence.

Because the HOEPS was a follow-up among previous study participants, our population is probably more motivated and the overall participation rates we observed are likely higher than would be expected in a survey within the general population. This structure, however, created an excellent opportunity for comparing participation between instruments: we had good contact information (both telephone and mailing), allowing our recruitment protocols to be similar between both instruments; all families were drawn from the same population of NBDPS participants and randomly assigned to an instrument, removing most potential confounding/bias in a comparison of the instruments; and we had demographic information on eligible non-participants, allowing us to evaluate differences in participation rates between specific subgroups. Our design may have been somewhat biased towards high participation rates among those assigned to the CATI, because participation in the NBDPS requires completing a telephone interview. All those eligible for the HOEPS therefore had telephone access and a demonstrated willingness to provide information over the telephone.

We found that participation rates were higher for both mothers and fathers assigned to the MQ versus the CATI. Participants assigned to the MQ also required fewer reminder call cycles or letters before completing the study versus participants assigned to the CATI. As we expected, the largest impact of survey method on participation rates occurred among control subjects. Case subjects are generally more highly motivated than control subjects, thus we would expect the attractiveness of a survey method to have less weight in motivating participation among cases than controls. In all demographic strata we examined, participation rates were higher for mothers assigned to the MQ versus the CATI. The distribution of participating subjects across demographic groups, however, was similar to the distribution of eligible subjects. This suggests that mothers recruited to the HOEPS are likely representative of NBDPS participants, and that the increased response to the MQ was not attributable to a specific demographic subgroup.

Future studies that evaluate the use of a MQ should consider whether the increased cost of postage and printing for a questionnaire will be offset by savings in recruitment costs and increased participation rates. We found that the MQ saved personnel time (primarily due to fewer reminders) compared to the CATI, while postage and printing costs of the MQ were only slightly increased compared to those for the CATI. Overall, the mailed questionnaire was more cost-effective for this study, though following up on missing or confusing responses to the MQ required considerable time and effort whereas interviewers could immediately probe vague answers during the CATI. Complex surveys soliciting detailed answers may therefore be better suited to a CATI than a MQ.

There are several important limitations to this study. The increased participation rates we observed among those invited to the MQ versus the CATI may not be generalizeable to all groups; our population consisted primarily of the parents of young children who might have difficulty finding quiet time to complete a telephone interview or predicting when they will have free time. A paper questionnaire might be easier for these busy parents to fill out a little at a time when opportunities arise. In this study, we were also unable to compare the impact of survey type on the validity of respondents’ answers. Research from the 1970s and 1980s found that responses to MQs were generally equivalent to [25] or more valid than responses to CATIs [24]. A 1979 study of item omission between mailed and telephone surveys found that participants were more likely to answer sensitive questions on a mailed survey [13]. These studies, however, were undertaken in decades past and it may not be accurate to apply these results to current survey research; few similar studies have been conducted recently. We also did not evaluate the impact of offering more than one mode of survey instrument, which has been shown to improve participation rates [26,27]. As internet coverage increases, web-based surveys may also provide a practical and economical alternative.

Despite these limitations, this study provides a valuable comparison of survey instruments. Since subjects were randomized to the MQ or CATI, neither self-selection effects nor confounding is likely to affect the comparison of the two instruments. This is supported by the similar distribution of maternal demographic characteristics between mothers assigned to each instrument, as well as the similarities we observed between all those eligible for HOEPS and those who completed participation. We obtained a higher response rate with less recruitment effort using the MQ.

## Conclusions

In a follow-up survey, we randomized families to be recruited to a MQ or a CATI. Both instruments contained the same information and similar recruitment protocols and materials. We obtained higher participation among mothers recruited to complete a MQ versus a CATI. This higher participation rate for the MQ held true in every demographic subgroup we examined. Although mothers who completed the MQ were less likely to give us permission to contact fathers, those fathers we contacted were more likely to participate compared to fathers recruited with the CATI; consequently we had higher overall participation from fathers using the MQ. While we found that it was very time-consuming to follow up on missing or confusing responses to the MQ, we also did not have to devote as much time to sending reminders for the MQ compared to the CATI. The cost-benefit ratio of one instrument versus another may vary depending on the target population and complexity of information being sought. Such information may guide other epidemiological studies, particularly those targeting the parents of young children, in deciding which mode of survey administration to implement.

## Competing interests

The authors declare that they have no competing interests.

## Authors’ contributions

PAR conceived the study. All authors assisted in designing the survey instruments and/or recruitment protocols for the HOEPS. CMR prepared all IRB documents, assisted in participant recruitment/ data management, and performed data analysis. SHS oversaw recruitment and data collection. All authors contributed to writing and revising this manuscript. All authors read and approved the final manuscript.

## Pre-publication history

The pre-publication history for this paper can be accessed here:

http://www.biomedcentral.com/1471-2458/12/579/prepub
